# Cassava Breeding I: The Value of Breeding Value

**DOI:** 10.3389/fpls.2016.01227

**Published:** 2016-08-29

**Authors:** Hernán Ceballos, Juan C. Pérez, Orlando Joaqui Barandica, Jorge I. Lenis, Nelson Morante, Fernando Calle, Lizbeth Pino, Clair H. Hershey

**Affiliations:** ^1^International Center for Tropical AgricultureSantiago de Cali, Colombia; ^2^Corporación Colombiana de Investigación AgropecuariaSanta Marta, Colombia

**Keywords:** within-family genetic variation, partial inbreeding, genetic gains, recurrent selection, additive genetic effects, non-additive genetic effects

## Abstract

Breeding cassava relies on several selection stages (single row trial-SRT; preliminary; advanced; and uniform yield trials—UYT). This study uses data from 14 years of evaluations. From more than 20,000 genotypes initially evaluated only 114 reached the last stage. The objective was to assess how the data at SRT could be used to predict the probabilities of genotypes reaching the UYT. Phenotypic data from each genotype at SRT was integrated into the selection index (SIN) used by the cassava breeding program. Average SIN from all the progenies derived from each progenitor was then obtained. Average SIN is an approximation of the breeding value of each progenitor. Data clearly suggested that some genotypes were better progenitors than others (e.g., high number of their progenies reaching the UYT), suggesting important variation in breeding values of progenitors. However, regression of average SIN of each parental genotype on the number of their respective progenies reaching UYT resulted in a negligible coefficient of determination (*r*^2^ = 0.05). Breeding value (e.g., average SIN) at SRT was not efficient predicting which genotypes were more likely to reach the UYT stage. Number of families and progenies derived from a given progenitor were more efficient predicting the probabilities of the progeny from a given parent reaching the UYT stage. Large within-family genetic variation tends to mask the true breeding value of each progenitor. The use of partially inbred progenitors (e.g., S_1_ or S_2_ genotypes) would reduce the within-family genetic variation thus making the assessment of breeding value more accurate. Moreover, partial inbreeding of progenitors can improve the breeding value of the original (S_0_) parental material and sharply accelerate genetic gains. For instance, homozygous S_1_ genotypes for the dominant resistance to cassava mosaic disease (CMD) could be generated and selected. All gametes from these selected S_1_ genotypes would carry the desirable allele and 100% of their progenies would be resistant. Only half the gametes produced by the heterozygous S_0_ progenitor would carry the allele of interest. For other characteristics, progenies from the S_1_ genotypes should be, at worst, similar to those generated by the S_0_ progenitors.

## Introduction

Most cassava breeding programs started in the 1970s or later. Ceballos et al. (2012) proposed that the initial progress was actually to finalize the domestication of the crop, i.e., to move from a crop adapted almost exclusively to rustic, low management conditions to one that responds well to more intensive management for productivity. By the 1990s officially released varieties had shown a significant increase (Kawano et al., 1998; Kawano, 2003) in fresh root yield (FRY) and dry matter content (DMC). An outstanding example is KU50, a variety released in Thailand in 1992, and still grown on more than 1 million ha annually in several countries in SE Asia. This variety, along with others released at about the same period, had a significant impact in the livelihoods of millions of resource-limited farmers (Kawano and Cock, 2005; Fu et al., 2014). It has been recently reported that selection alters the relationship between FRY and DMC. The selection process favors genotypes with high dry matter productivity through either high FRY or high DMC, but it is very difficult to find genotypes that are outstanding simultaneously for both traits (Ceballos and Hershey, 2016).

However, the impressive genetic progress achieved from 1975 to 1995 has slowed down considerably in the last two decades (1995–2015). Combined analyses of different reports from cassava breeding in Thailand indicate that gains from 1995 to 2015 are at best half of those observed in the previous two decades for FRY and DMC (CIAT, 2007; Ceballos and Hershey, 2016). Similar trends can be observed in Colombia and Brazil.

It was expected that biotechnology tools, such as marker assisted selection, would help recover the rate of genetic gains. Molecular biology has been successful in diagnostics for cassava diseases and their genetic diversity (Restrepo and Verdier, 1997; Hernández Pérez et al., 1999; Monger et al., 2001a,b; Álvarez et al., 2003, 2009; Calvert et al., 2008; Legg et al., 2011); gene expression studies in host-pathogen interactions (Hong and Stanley, 1995; Fregene et al., 2004; Kemp et al., 2004, 2005); introgression of resistance to cassava mosaic disease (CMD) in Latin American germplasm (Egesi et al., 2006; Okogbenin et al., 2007), or dissection of the pathway leading to post-harvest physiological deterioration in cassava roots (Reilly et al., 2007). The first molecular map of cassava was first published two decades ago (Fregene et al., 1997). Yet, the only successful applied experience of marker assisted selection in cassava breeding to date has been for resistance to CMD (Fregene et al., 2000; Akano et al., 2002; Rabbi et al., 2014), while impact on increasing FRY has been limited.

In spite of these advances in breeding tools, the slowing down in genetic gains for FRY has not been reversed. Breeders continue to aim for high yield, but have also shifted attention to other value-added traits that are easier to breed such as nutritional quality (Ceballos et al., 2013; Maziya-Dixon and Dixon, 2015), starch functional properties (Aiemnaka et al., 2012) or resistance to CMD (Rabbi et al., 2014).

Cassava breeders typically apply phenotypic recurrent selection, as is common for clonally propagated crops (Burton, 1992; Grüneberg et al., 2009; Lebot, 2010; Quero-García et al., 2010; Ceballos et al., 2012). Because of the low multiplication rate of cassava from stem cuttings, it takes several years to have enough planting material available for replicated multi-location evaluations, under conventional propagation systems (Ceballos et al., 2004, 2012). A typical selection cycle requires 2 years to produce the progeny (botanical seeds) of planned crosses and 6 consecutive years of field evaluation. Initial phenotypic evaluations are based on unreplicated trials grown in one or, at most, two locations. Critical selection decisions need to be taken during this lengthy process: breeders try to reconcile the practical need to reduce the large number of genotypes in the early stages of selection with the awareness that selection based on unreplicated trials is prone to large experimental errors.

Ceballos and co-workers suggested the possibility of using breeding value (e.g., general combining ability) for cassava genetic enhancement based on promising results they had observed using phenotypic data (Ceballos et al., 2004). Falconer (1981) defined breeding value of an individual as the mean value of its progeny, a simple yet powerful concept in plant and animal breeding. The breeding value is the deviation of the progeny generated by a given progenitor from the average of a reference population. Breeding value depends on the average performance of the reference population as well as on the value of the alleles that each progenitor can transfer to its progeny (Falconer, 1981). Typically, breeding value is related to additive genetic effects, although some dominance effects (e.g., a single dominant source of resistance to a given disease or pest) can influence breeding values. Best linear unbiased prediction (BLUP) was originally developed for more accurate estimation of breeding values in animal breeding and has now been widely used in many areas of research including different crops (Henderson, 1975; Pander and Allen, 1995; Bernardo, 2002). However, it seems that it has not gained the same popularity in plant breeding (Piepho et al., 2008). Genomic selection currently under pilot testing in cassava brings hope of a positive impact for enhanced productivity (de Oliveira et al., 2012; Wolfe et al., 2016) and evolved from earlier applications of BLUP (Heffner et al., 2009). Genomic selection is a form of marker assisted selection that sorts individuals out, based on genomic estimated breeding values (Nakaya and Isobe, 2012). Genomic selection relies on the estimation of breeding values for quantitative traits based on whole genome genotypes through the simultaneous estimation of marker effects in a single step (Heslot et al., 2012).

The current study consolidates phenotypic data from 14 years of successive trials in a sub-humid tropical environment of Colombia, from more than 20,000 genotypes initially evaluated in single row trials—SRT. The data consolidated, curated and organized for analysis can be accessed at http://dx.doi.org/10.7910/DVN/QB9FUW. The original raw data is also available at https://www.cassavabase.org. The main objectives were, (i) to estimate breeding values of progenitors of the more than 20,000 genotypes initially evaluated; (ii) assess the usefulness of these breeding values for predicting which genotypes eventually reach the most advanced stage of selection (uniform yield trials—UYT), grown in several locations and years, and (iii) attempt to identify factors that affect the probability of clone(s) from a given progenitor to reach the UYT stage.

## Materials and methods

### Breeding objectives and selection criteria

Breeders apply a wide range of objectives in cassava in response to the diversity of production environments, management practices, and end uses. However, only a few are broadly accepted as common key traits for improvement: FRY; high and stable DMC; suitable plant architecture, and resistance to locally or regionally relevant pests and diseases. At CIAT, in addition to individual ratings, breeders integrate plant architecture and resistance to biotic/abiotic stresses into a single score indicating overall desirability of the above-ground plant appearance (plant type score or PTS) where 1 is very good and 5 is very poor. Because of the low heritability of FRY in early stages of selection, cassava breeders for many years have applied indirect selection for yield by using correlated traits with higher heritabilities, such as harvest index (HIN) (Kawano et al., 1998).

CIAT generally applies a selection index (SIN) that integrates these four relevant variables, assigning them best-judgment weight (in italics in the formula below) established by the breeder's experience (Ceballos et al., 2012):

$$\begin{array}{l} \text{SIN}=\left(\text{FRY}{\;}^{*}\;10\right)+\left(\text{DMC}{\;}^{*}\;10\right)-\left(\text{PTS}{\;}^{*}\;5\right)+\left(\text{HIN}{\;}^{*}\;3\right) \end{array}$$

In the case of PTS the desired target is a lower score. Therefore, a negative sign is assigned to the respective term in the SIN equation.

### Evaluation and selection process

**Figure 1:**
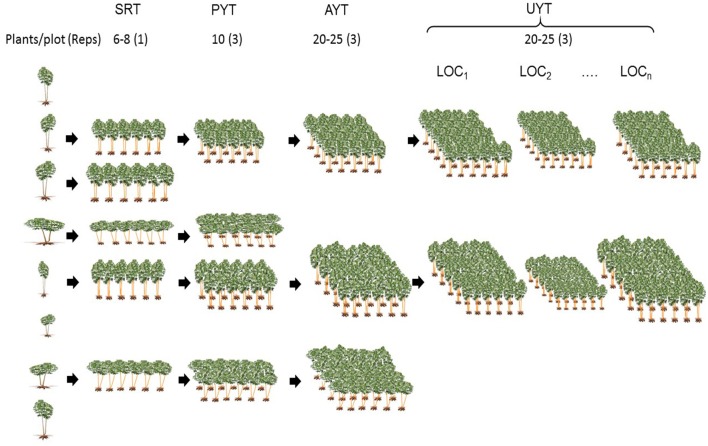
**Illustration of the different stages of a typical evaluation process in cassava breeding**. Plants from germinated seed (seedling plants) are grown in the field and used as the source of clonal planting material (left side). The first evaluation takes place in single row trials (SRT), followed by preliminary (PYT) and advanced (AYT) yield trials. The first multi-location evaluation is in the uniform yield trials (UYT), or sometimes earlier, in the AYTs. Size of plots in UYT has been slightly modified to illustrate the effect of different environments on the growth of cassava.

We obtained botanical seed by controlled (full sibs) or open (half sibs) crossing among outstanding progenitors (all cassava genotypes currently used in breeding are heterozygous). Seed was germinated, seedlings grown for about 2 months in a greenhouse, and then transplanted to the field. The seedling plants (F_1_) were grown in Palmira, Valle del Cauca, Colombia (CIAT headquarters), which offers fertile soils, moderate temperatures and availability of irrigation—ideal for high cassava productivity. Selection and harvest of plants took place at 9–10 months after transplanting. The only selection criterion applied was the capacity of the plant to produce eight vegetative cuttings (20 cm stem pieces) for the following stage of selection. This step initiated the long process of phenotypic recurrent selection as described below (Figure 1).

#### Clonal evaluation trials or single row trials (SRTs)

This is the first stage where selection for agronomic performance takes place in the sub-humid environment (Caribbean coast of Colombia). The region is characterized by moderate rainfall (800–1200 mm annually) and a long dry season (3–4 months), typical of many cassava-growing regions of the world. Trials usually include about 1000–2000 genotypes, each represented by six to eight plants in a single row (1–2 ha), in a single location. About 150–250 genotypes are selected for the next stage of evaluation. An important feature of SRTs is that, being the first stage of the selection process, information from all progenies (selected or not) is available, thus providing unbiased information about the progenitors used to generate them.

#### Preliminary yield trials (PYTs)

Each genotype is represented in three repetitions with 10-plant plots (two rows of five plants). A randomized complete block design is used in all remaining type of trials. All plants in each plot (except the front plant in each row) are harvested. PYTs are planted in a single location.

#### Advanced yield trials (AYT)

Plots consist of four (or five) rows and five plants per row, with, three replications. The six (or nine) central plants are harvested to generate the data used in the selection process. AYTs are usually planted in a single location.

#### Uniform yield trials (UYT)

This is the final stage in the CIAT-managed evaluation and selection process. Plot size, number of repetitions and planting arrangement is the same as those for AYTs. UYTs are planted for 2 consecutive years in 5–10 locations. Typically UYTs will have 20–25 experimental clones and 5–8 local or commercial checks. Farmer and end user criteria are used during each step of selection, and they are invited to participate for more intensive input and interaction with breeders during the harvest of AYTs and UYTs. In addition, planting material of the most promising clones is shared with key farmers for semi-commercial evaluation. In general, varieties are released by national programs only after successful performance (according to the farmers' and end-users' criteria) in these semi-commercial evaluations (0.5–1.0 ha).

### Data analysis

Data from evaluation trials conducted from 2000 through 2013 were used. Target growing conditions included various sites within the sub-humid environment, the most important cassava growing region in Colombia and in most of the world.

This large database was prepared for analysis with SAS (2008). The first step was to consolidate data from different trials grown during successive years into a large megafile. A total of 1038 full- or half-sib families were evaluated in these trials involving a total of 20,229 genotypes evaluated in the SRTs (9108 from full-sib families and 11,221 from half-sib families). Four variables (FRY, DMC, PTS, and HIN) were considered for the analysis and used to estimate a selection index (SIN) for each genotype, using the same weights as in the formula described above. SAS Proc Means procedure was used to obtain the family averages for every trait, including selection index.

From the initial number of genotypes evaluated in SRT, 2652 were selected and evaluated in PYT, 567 in AYT and only 114 in UYT. This study concentrates on the data from the first and last stages of selection (SRT and UYT, respectively) and will not consider the intermediate stages.

Data from all the individual genotypes belonging to a given full- or half-sib family was consolidated to obtain the respective averages and other statistical parameters for the key variables: FRY, DMC, PTS, HIN, and SIN. Since progenitors are used to generate more than one family, averages for each progenitor across all the families that it had generated were estimated. The phenotypic average of all the progenies (across different families) generated by a given progenitor will be considered as the breeding value of that progenitor. Phenotypic data from the 9108 full-sib genotypes was used twice: for the estimation of breeding values of the progenitor used as female, and for the breeding value when used as a male.

Data from each progenitor was not balanced because of lack of a uniform number of progenies evaluated from each progenitor. The number of crosses (e.g., full- or half-sib families) generated from each progenitor was also variable, as was the number of years in which progenies from a given progenitor were involved. It is acknowledged therefore that breeding value as estimated in this study is not as accurate as that obtained, for instance, from a diallel study. However, the estimated breeding values fully agree with the original concept in Falconer described earlier, and are based on actual data generated by an ongoing breeding process.

Progenitors represented by fewer than 50 genotypes among the progeny, across all families in which they had been used, were discarded from the analysis. A sample size of <50 individuals was considered too small to properly represent the breeding value of the respective progenitor. The initial number of progenitors (297) was therefore reduced to 107.

## Results

A large dataset was consolidated from the different trials conducted from 2000 to 2013. A total of 20,229 genotypes were evaluated in SRT. Table 1 provides a general description of the 107 progenitors analyzed in this study (after discarding those represented by progenies with fewer than 50 clones). The average size of the progenies from the 107 progenitors was 255. There was wide variation in the sample size for each progenitor (ranging from the minimum required of 50 progenies all evaluated in a single year, through 1350 progenies evaluated across the 14 SRTs). This variation in the number of progenies from each progenitor relates to the highly variable flowering behavior of different cassava genotypes (Ceballos et al., 2012). Some genotypes may flower 3–4 times during a year, whereas others flower only once. In few cases plants may have to be grown for more than a year for them to flower for the first time.

**Table 1:** **Progenitors (107) selected in the study and number of progenies (#) derived from them**.

**Progenitor**	**#**	**Progenitor**	**#**	**Progenitor**	**#**	**Progenitor**	**#**	**Progenitor**	**#**	**Progenitor**	**#**
SM1565-17	1350	SM2773-32	472	C-4	233	SM2775-4	126	GM273-57	92	CM6756-15	68
TAI8	1255	SM1521-10	445	CM4843-1	232	SM1068-10	125	SM1600-4	89	SM2615-25	67
SM1665-2	915	CM4365-3	437	SM737-38	230	CM7514-7	124	SM2619-6	87	SM2772-8	67
CM8027-3	906	KU50	425	SM1427-1	215	SM2621-4	118	VEN167	85	BRA496	65
SM1411-5	861	SM2081-34	418	CM9456-12	211	CM7389-9	117	SM3058-29	79	CM7395-5	65
SM1219-9	795	SM2546-40	403	CM8475-4	210	SM2621-29	115	SM2545-20	78	BRA1107	64
SM805-15	760	SM2772-5	403	SM2619-4	210	SM2546-52	114	SM2923-3	78	SM1152-13	64
CM6754-8	735	SM2620-1	334	SM1778-45	206	SM2769-11	114	SM494-2	77	SM2772-2	63
SM1433-4	718	CM7985-24	326	CM9560-1	204	GM290-50	109	GM259-167	75	C-243	62
CM7514-8	667	SM1789-20	295	SM1210-10	180	CM3306-4	108	SM1282-2	75	CM4574-7	58
CM6758-1	663	SM2546-32	294	TAI1	175	SGB765-2	108	R90	73	COL945	58
CM9067-2	657	SM2780-17	291	SM1637-22	165	SM2775-2	105	SM2546-54	73	CT20-2	54
SM1511-6	647	SM1759-29	290	SM1656-7	159	GM462-4	103	CG1141-1	72	SM1650-7	54
CM523-7	584	SM2629-36	272	SM890-9	155	SM1669-5	103	CM9924-6	72	CM3372-4	52
SM2192-6	536	NGA19	266	SM1201-5	152	SM1669-7	98	CM6756-13	71	SM2619-1	51
SM2782-4	519	SM1657-12	264	SGB765-4	151	SMB2446-2	98	SM2623-1	71	VEN25	51
SM1438-2	489	SM2545-22	240	SM1422-4	129	SM1973-25	93	CM3555-6	69	CM9912-107	50
CM2772-3	481	C-18	235	SM643-17	128	SM2603-9	93	CM7951-5	69		

A total of 114 genotypes were evaluated in different UYTs in the sub-humid environment during the 2000–2013 period. A key objective of this study was to identify factors that influence the probability of clone(s) from a given progenitor to reach the UYT stage, taking into consideration that, in vegetatively propagated crops, breeding values can be measured across generations with the same genotypes. Progenitors of the 114 clones that reached UYTs were therefore identified. Only three progenitors (CM4919–1, CM681–2, and SM1565–15) of clones in UYTs were not included in the study because they were represented by fewer than 50 progenies. The progenitors of clones reaching the UYTs that are analyzed in this study are listed in Table 2, along with the number of clones derived from them which reached that stage. There was a large variation in the number of clones in UYTs representing different progenitors. Twenty clones in UYTs had been derived from SM1411–5, suggesting that this progenitor has excellent breeding value. Similarly SM 1665–2, CM 8027–3, CM 9067–2, and CM 7514–8 were progenitors of at least 10 genotypes evaluated in UYTs. On the other hand, 12 progenitors were represented only once by their progenies in UYTs and 66 progenitors were not represented in UYTs at all. Results suggest, therefore, that there were strong differences in the probabilities of progenies from a given progenitor reaching the last stage of selection (Table 2). From the breeding point of view, it would be very useful to explain why progenies from SM1411–5, for example, had a higher chance of reaching the last stage of selection and, conversely, why so many progenitors failed to contribute with any clones in UYTs.

**Table 2:** **Progenitors (41) of clones that reached the UYT and were represented by more than 50 progenies in the SRT; and number of clones (#) from each of these progenitors**.

**Progenitor**	**#**	**Progenitor**	**#**	**Progenitor**	**#**	**Progenitor**	**#**	**Progenitor**	**#**	**Progenitor**	**#**
SM1411-5	20	TAI8	7	SM1759-29	4	KU50	2	SM2629-36	2	SM1210-10	1
SM1665-2	15	SM1521-10	6	SM890-9	4	SM1422-4	2	SM737-38	2	SM1637-22	1
CM8027-3	12	SM1438-2	5	CM523-7	3	SM1511-6	2	CG1141-1	1	SM1657-12	1
CM9067-2	12	SM2192-6	5	SM1219-9	3	SM1656-7	2	CM4574-7	1	SM2081-34	1
CM7514-8	10	SM805-15	5	SM1565-17	3	SM1669-5	2	CM7395-5	1	SM2773-32	1
SM1433-4	8	CM6754-8	4	CM7985-24	2	SM1778-45	2	NGA19	1	SM643-17	1
CM4365-3	7	SM1427-1	4	CM8475-4	2	SM1789-20	2	SM1201-5	1		

**Figure 2:**
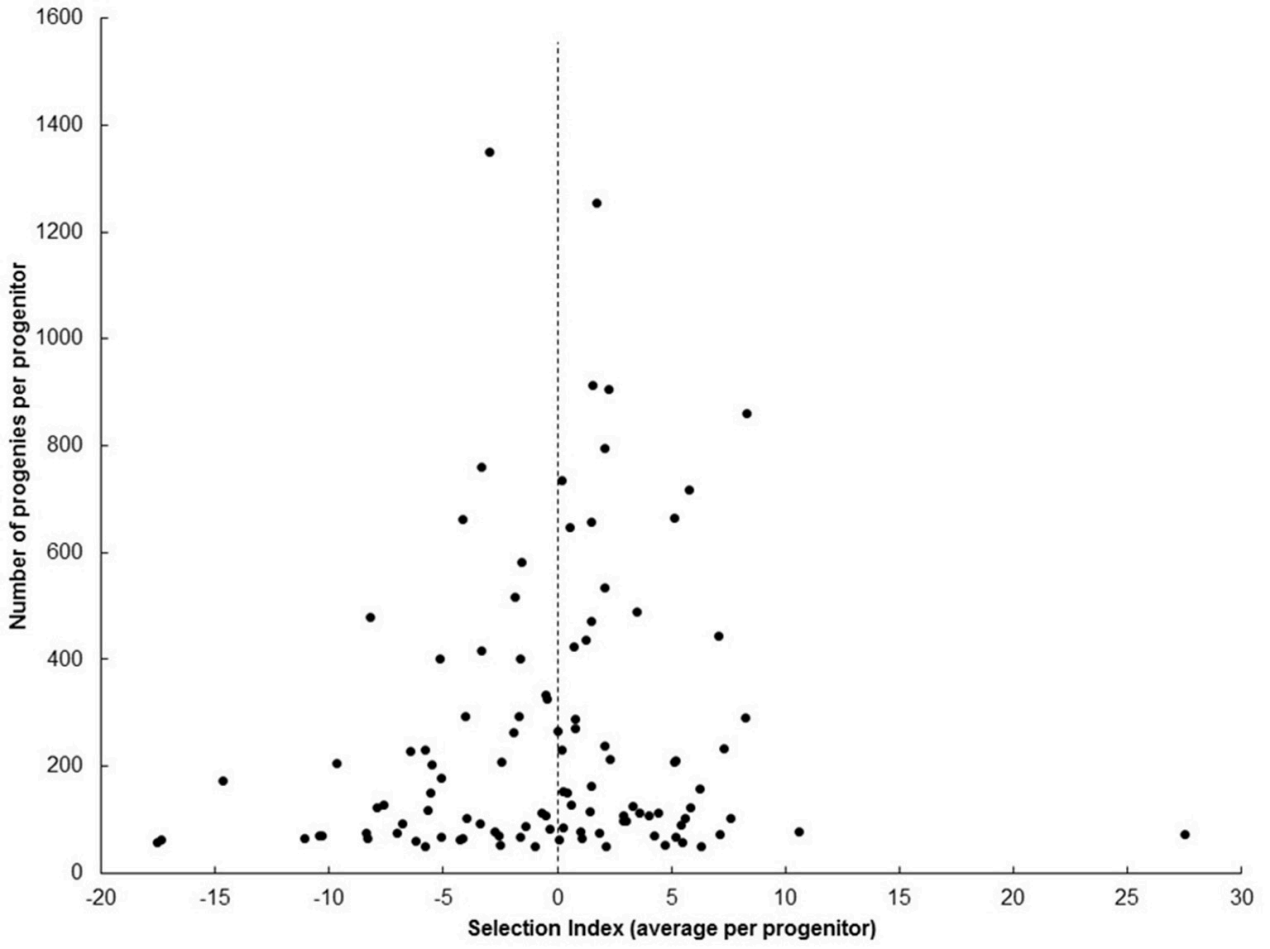
**Relationship between average selection index (SIN) of each progenitor with the respective number of clones representing them at UYT**. Extreme average SIN were observed for progenitors represented by fewer than 200 progenies.

Selection from SRT, through PYT, AYT, and UYT is based on the SIN that integrates the information of four key variables (FRY, DMC, HIN, and PTS). If the selection index is formulated well, average SIN for the progenies of each progenitor should be the parameter most closely associated with the true breeding value of each progenitor measurable at SRT. Table 3 presents the best and worst ten genotypes, based on the average SIN of their progenies from SRTs. Data from SRTs was used because it takes into consideration information from all progeny derived from a given progenitor, regardless of whether or not they were selected. Average SIN (≈breeding value) of the progenies from SM1411-5 was ranked third-best among the 107 genotypes analyzed and was represented by 861 progenies (a very robust progeny size). Figure 2 presents the relationship between average SIN from each progenitor and the size of their respective progenies. Smaller samples tend to show more extreme variation (e.g., ranging from very high to very poor breeding values), compared to larger samples. This is not surprising as standard deviations of the mean and sample sizes are inversely associated (Steel and Torrie, 1988). The information presented in Figure 2 indicates that breeding value (estimated as average SIN for the progenies of each progenitor) is heavily influenced by the size of the progenies rather that the genetic merit of each progenitor: extreme cases (positive or negative) were only found for progenitors represented by fewer than 200 progenies.

**Table 3:** **Average selection index (SIN) of the 10 best and 10 worst progenitors, minimum and maximum SIN, as well as the size of their respective progenies**.

**Progenitor**	**Size**	**SIN (Average)**	**SIN (Minimum)**	**SIN (Maximum)**
R90	73	27.5	−3.9	58.3
SM2545-20	78	10.6	−31.8	52.8
SM1411-5	861	8.3	−76.7	46.1
SM2780-17	291	8.3	−47.5	53.0
GM462-4	103	7.6	−55.8	43.3
C-18	235	7.3	−45.9	52.6
SM2546-54	73	7.1	−41.0	36.8
SM1521-10	445	7.0	−54.7	48.6
SM2619-1	51	6.3	−26.7	30.2
SM1656-7	159	6.2	−58.8	52.8
CM2772-3	481	−8.2	−63.9	33.1
SM2615-25	67	−8.4	−51.7	19.9
SM494-2	77	−8.4	−51.7	22.4
SM1778-45	206	−9.7	−69.4	36.0
CM6756-13	71	−10.3	−48.0	31.6
SM2623-1	71	−10.4	−53.0	40.0
BRA496	65	−11.1	−46.0	23.0
TAI1	175	−14.7	−68.9	35.4
SM2772-2	63	−17.4	−86.6	27.7
COL945	58	−17.6	−48.1	17.7
Average	255	−0.46	−55.49	41.30

*The list is ordered from higher to lower average SIN*.

**Figure 3:**
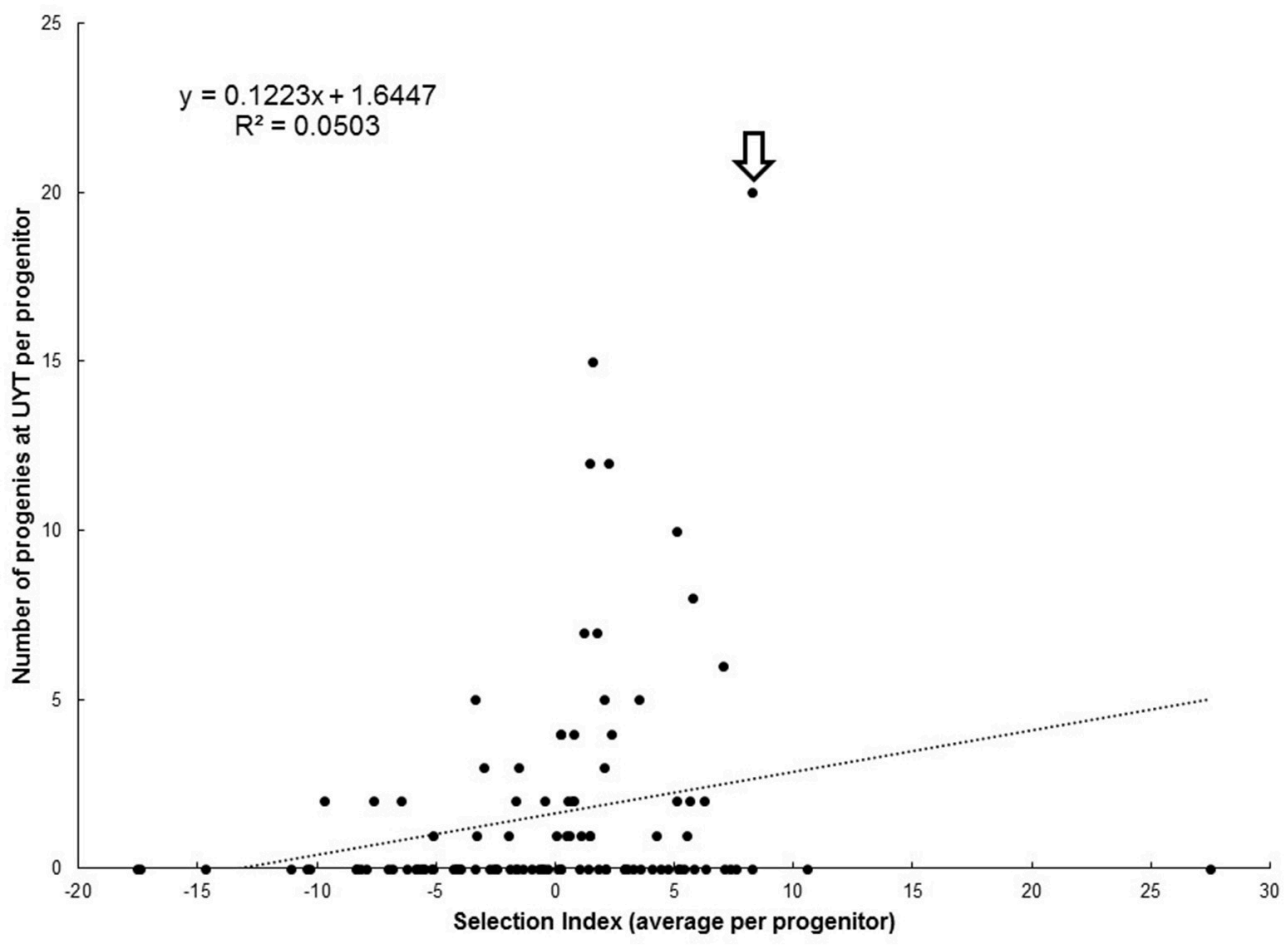
**Relationship between average selection index (SIN) of each progenitor with the respective number of clones representing them at UYT (arrow identifies SM 1411-5)**.

Figure 3 illustrates the relationship between average SIN for each progenitor and the number of their respective progenies reaching the UYT stage. The performance of SM 1411–5 is worth highlighting because it was a progenitor in about 20% of the clones reaching the UYT and its average SIN was 8.3, suggesting an association between high and positive SIN and success in deploying progenies in UYT. On the other hand, several progenitors with average SIN above 10 had no clones representing them in UYTs. The regression of number of clones in UYT on average SIN for each progenitor (Figure 3) shows a negligible *r*^2^ = 0.05, indicating that breeding value (e.g., average SIN for each progenitor) is not a good predictor of the probabilities of a clone from a given progenitor reaching the UYT.

In addition to the average SIN values, Table 3 provides the maximum and minimum SIN for the individual clones derived from each progenitor. Maximum SIN values are very relevant because they identify the best genotypes which should be, ultimately, those reaching UYT. One of the problems cassava breeders face is the huge within-family variation arising from the fact that progenitors are heterozygous. That variability (illustrated by the wide range of variation of individual SINs in Table 3) weakens the identity of families and supports the idea that outstanding hybrids can be obtained basically from each and every family (Losada Valle, 2015).

**Figure 4:**
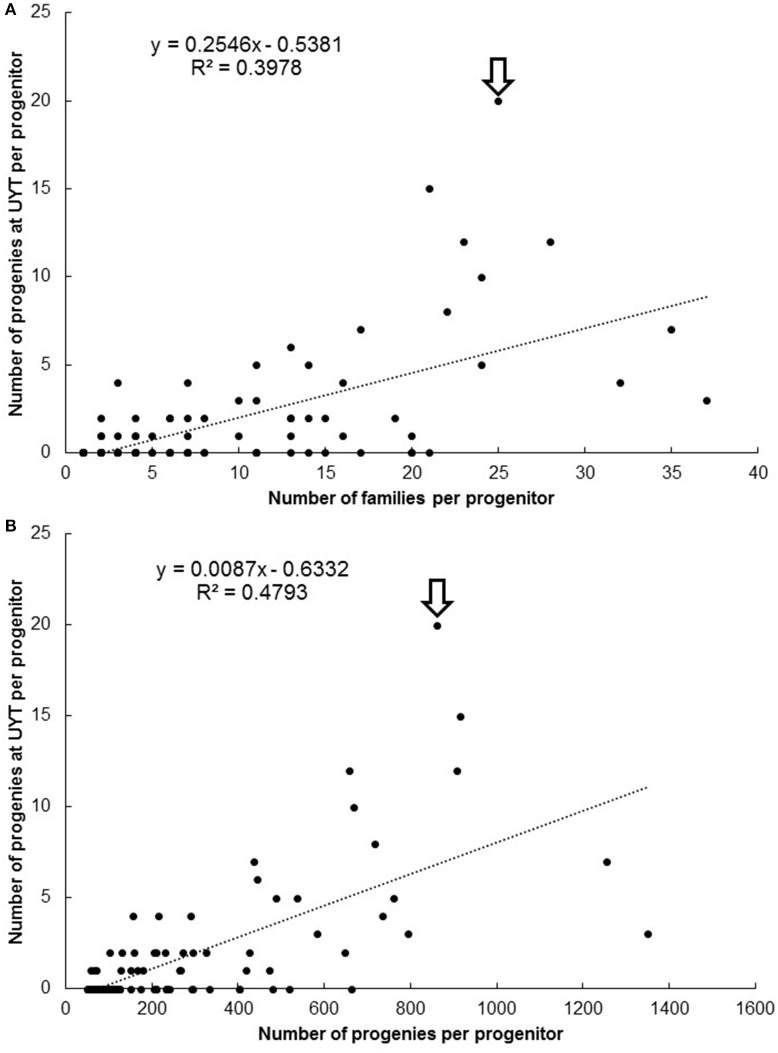
**Number of families (A) and progenies (B) per progenitor against the number of clones derived from each progenitor reaching UYT (arrow identifies SM 1411-5)**.

The plots presented in Figure 4 describe the relationship between number of families (Figure 4A) and progenies (Figure 4B) per progenitor against the number of clones derived from each progenitor reaching UYT. The *r*^2^ value from the regression analysis of number of progenies from a progenitor reaching UYT on the total number of progenies per progenitor (0.48) was considerably better than the same parameter from a regression based on average SIN in Figure 3 (0.05). Number of families generated by each progenitor was also a better predictor (*r*^2^ = 0.40) than average SIN of the probability of its progeny reaching UYT.

**Figure 5:**
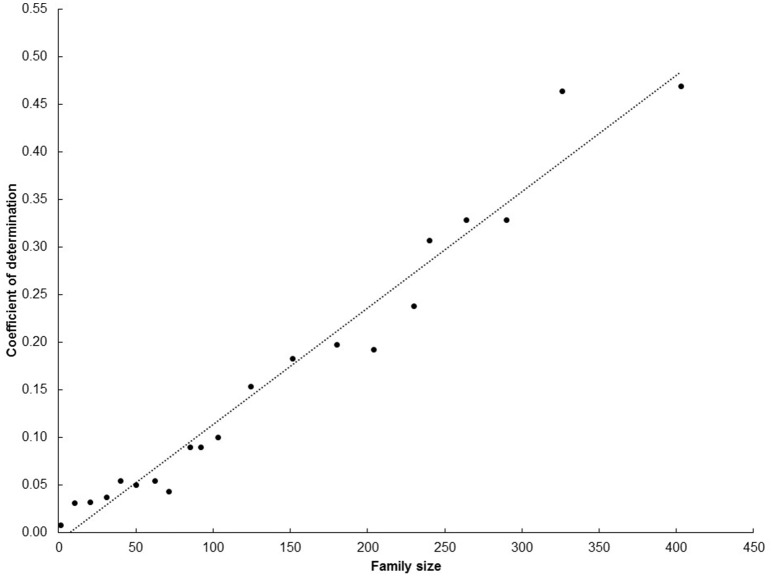
**Coefficient of determination for the regression of average SIN at SRT on number of progenies reaching the UYT stage considering different family sizes**.

It is clear that family size, as expected, strongly influences the results of this study. The initial analysis arbitrarily set a minimum family size *n* = 50. This number was a reasonable starting point (it was rendered to be large enough to properly represent the breeding potential of each progenitor, but not too large to reduce the total number of progenitors analyzed) but, nonetheless it was arbitrary. Therefore, an exercise was made to analyze the relationship between average SIN at SRT and the probability of progenies reaching UYTs using different family sizes. Figure 5 presents the results of this exercise. The coefficient of determination increased linearly from negligible (when family size < 50) to values larger than 0.25 (when family size > 250). Family size > 300 provided much larger coefficient of determination (>0.45). Results presented in Figure 5 make sense: larger samples of progenies from a given progenitor are expected to provide more reliable information than smaller samples. It is not surprising that a large family size (e.g., 250 genotypes) is required to somewhat predict the chances of one of its members reaching the UYT stage. This is a reflection of the large within-family genetic variability generated from the heterozygous progenitors used in cassava breeding (Ceballos et al., 2015). Families larger than 400 were not considered as the number of progenitors that met this requirement would have been drastically reduced.

## Discussion

This study focuses on data from the extremes of the selection process—from the earliest (SRT) to the last stage (UYT). Between these two steps, however, are the PYT and AYT stages. It has been suggested that the phenotypic performance of individual genotypes may “evolve” through the different stages of selection. Epigenetic effects and the impact that biotic and abiotic factors have in the quality of planting material may affect differentially the performance of different genotypes through time (Ceballos and Hershey, 2016; Joaqui et al., in review). This can partially explain the poor association between average SIN at SRT and probabilities of a progenitor being represented in UYT depicted in Figure 3. The large within-family genetic variation in cassava is another factor explaining that poor association (Supplementary Image). The implementation of new genomic tools can contribute to our understanding of the differences in breeding values suggested by data in Tables 2, 3. For the implementation of genomic selection, however, it would be advisable to use phenotypic data from later stages of selection, once the phenotypic performance of each genotype has “stabilized.”

Phenotypic recurrent selection in cassava has the advantage that the cloned genotypes can be evaluated and selected many times in different locations and growing seasons. The gradual selection, through four different stages (SRT, PRY, AYT, and UYT) allows the selection of genotypes that have shown consistently outstanding performances. Data from SRT is of particular relevance because it offers unbiased information about the progenitors, i.e., data from all progenies, selected or not. Although SRT data is prone to large experimental errors (single plot at one location and usually large environmental variation in the evaluation sites), averages across many genotypes tend to provide more robust information.

Selection of progenitors based on their general combining ability or breeding value in cassava, originally proposed by Ceballos et al. (2004) is further supported by the large variability in number of clones at UYT representing each progenitor (Table 2). It is clear that certain genotypes are better progenitors than others. The fact that 20 out of 114 clones reaching UYTs were derived from SM 1411–5 is a convincing evidence for this statement. SM 1665–2; CM 8027–3; CM 9067–2; CM 7514–8, SM 1433–4, CM 4365–3; and MTAI 8 also were well represented by their progenies in UYTs. However, the average SIN from these progenitors was not outstanding (they were not among the best 10 progenitors), except for SM 1411–5 (Table 3). On the other hand, the average number of progenies from all these genotypes was 802 (ranging from 437 to 1255), well above the average across all progenitors (255). The best predictor for the probability of the progeny of a given clone to reach the UYT seems to be the number of progenies derived from it that are evaluated in SRTs. This is of little help for breeders. It is recognized that the large variation in the number of progenies evaluated from each of the progenitor in this study is a weakness. On the other hand, this reflects the dynamics in any cassava breeding program. It is easy to obtain botanical seed from certain clones and difficult from others. The reproductive biology of cassava will prevail over efforts made to balance the number of progenies from each genotype. The ongoing research to develop a protocol for the induction of flowering (Next Generation Cassava Breeding project, www.nextgencassava.org) will facilitate achieving a more balanced number of progenies from each progenitor.

The idea that “good hybrids can be obtained from almost every family” (assuming that parents are basically adapted to the broad biotic and abiotic conditions of the target environment) arises from the large within-family segregations that breeders observe in their nurseries, particularly for traits such as FRY. It is this large within-family variation, however, that weakens the usefulness of breeding value in cassava. It is the best clone(s) within each family that may eventually reach UYTs and it is the identification of that particular clone that is difficult and expensive. The use of homozygous progenitors in cassava would lead to a reduction of within-family genetic variation, in fact to zero unless there existed some residual heterozygosity (Ceballos et al., 2015). However, it is currently difficult to produce inbred genotypes in cassava. Successive self-pollinations are time consuming and favor the selection of early flowering genotypes with profuse branching architecture. Progress in the development of a protocol for the production of doubled haploids has been made (Perera et al., 2013) but is not yet routinely feasible. In the meantime, an alternative option is the use of partially inbred progenitors (e.g., S_1_ or S_2_ genotypes). This approach would reduce considerably the within-family genetic variation and in turn help breeders to more easily identify the true breeding value of these progenitors. Inbreeding depression is prevalent for FRY but not so much for traits such as plant height and traits related to above ground biomass (Rojas et al., 2009; Kawuki et al., 2011; de Freitas et al., 2016).

**Figure 6:**
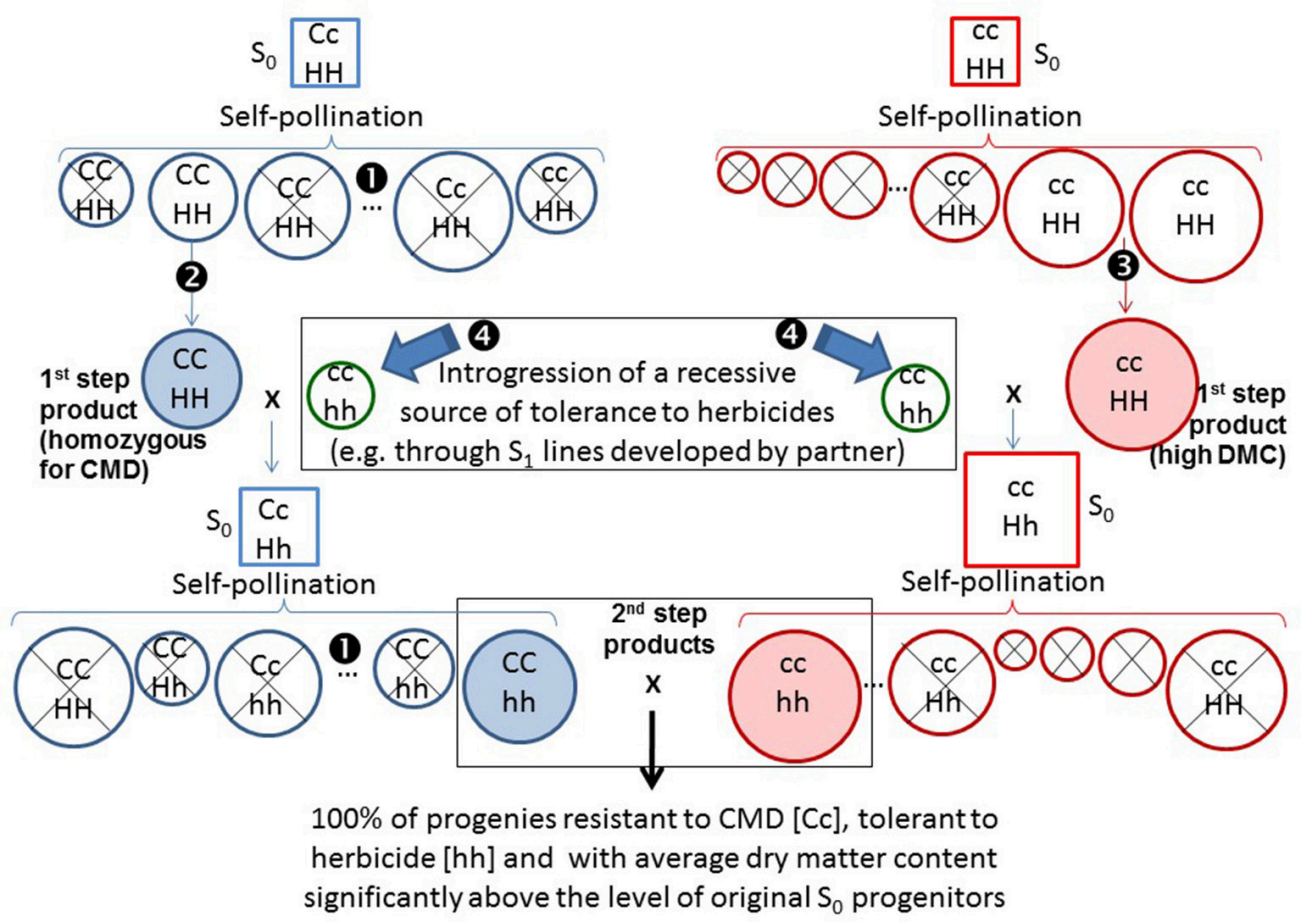
**Illustration of the way breeding value could be consistently improved in a stepwise fashion in two “***complementary”*** breeding populations**. Squares are used for S_0_ genotypes, whereas circles are used for partial inbreds. On the left, selections are made for resistance to CMD. Molecular markers can be used to distinguish homozygous [CC] from heterozygous [Cc] genotypes (❶). In addition to homozygous resistance to CMD, segregating S_1_ genotypes are selected for agronomic performance (❷). Diameters of the circles (or size of squares for S_0_) in both left and right diagrams represent levels of DMC (larger circles or squares, higher DMC). On the right, selections in the “*complementary”* population are made for increased dry matter content (❸). This population does not carry resistance to CMD so the genotype for this trait [cc] has not been included in every genotype. The selected products (S_1_ genotypes) from these first steps of selection are shaded. Both products, however, are susceptible to a target herbicide. In a parallel process (perhaps from a partner), S_1_ genotypes homozygous for a recessive source for tolerance to a herbicide have been generated (❹). The S_1_ genotypes selected for resistance to CMD or high DMC are then crossed with the source of tolerance to herbicides. The resulting crosses will be heterozygous for monogenic traits and intermediate for DMC. Self-pollination of the resulting crosses will allow the recovery of S_1_ genotypes that are homozygous for CMD and for tolerance to the herbicide (left side), or have improved levels of DMC combined with tolerance to the herbicide (right side). The second-step products are also shaded. Crossing the second-step products generate progenies that are 100% resistant to CMD [Cc], and tolerant to the herbicide [hh] and have excellent levels of DMC.

Partial inbreeding would not only contribute to identifying more clearly the breeding value of progenitors but it could also be the way to improve it (Kaweesi et al., 2016). Figure 6 illustrates this concept. For example, resistance to CMD has been linked to a single dominant factor (Rabbi et al., 2014). If an S_1_ genotype homozygous for the resistance to CMD was used (CC in Figure 6) instead of the (putatively) heterozygous S_0_ progenitor from which it was derived, its breeding value would double (e.g., 100% of the progenies rather than 50% of the progenies would be resistant to CMD). This concept is described on the left side of Figure 6. In addition to homozygous resistance to CMD, segregating S_1_ genotypes would be selected for agronomic performance as well. Similarly a “*complementary”* breeding population may be developed for increased levels of DMC (right side of Figure 6). The idea of “*complementary”* populations has been successfully implemented in commercial vegetables breeding (Knapp, personal communication). One population for example can be the source for defensive traits, while the other would provide desirable quality traits to the resulting hybrids.

Let's assume that a new recessive source of tolerance to a given herbicide has been identified. The source of tolerance is already partially inbred and homozygous (hh) for tolerance to the herbicide. The initial products (e.g., S_1_ genotypes) from the first step of selection in the two complementary populations presented in Figure 6 are susceptible (HH) to the herbicide. The S_1_ genotypes selected for resistance to CMD or high DMC are then crossed with the source of tolerance to herbicide. The resulting crosses will be heterozygous for the monogenic traits and intermediate for DMC. Self-pollination of the resulting crosses will allow the recovery of S_1_ genotypes that are homozygous for CMD and for tolerance to the herbicide (left side of Figure 6) or have improved levels of DMC combined with tolerance to the herbicide (right side of Figure 6). Crossing among the second-step products generates progenies that are 100% resistant to CMD (Cc), tolerant to the herbicide (hh), and have excellent levels of DMC.

The key principle here is that the gametes produced by the selected S_1_ genotypes should carry a higher frequency of desirable alleles. This is clearly the case for the traits these genotypes had been selected for (e.g., resistance to CMD). For other traits the frequency of desirable alleles at worst should be (on average), similar in the S_1_ genotypes and in the S_0_ progenitors from which they were derived. More likely, however, for other traits the frequency of desirable alleles should be higher because deleterious factors (e.g., albino plants) exposed in partially inbred genotypes would be eliminated. Crosses among the selected partially inbred lines, because of their enhanced breeding value, will generate (on average) better performing hybrids. A second and fundamental advantage of the proposed scheme is that it allows for the gradual, consistent, stepwise fixation of simply inherited traits in the partially inbred selected genotypes. Eventually, partially inbred lines from different heterotic groups (when identified or developed) would allow the implementation of conventional reciprocal recurrent selection schemes.

There are several traits in cassava that have relatively simple inheritance and would be easy to fix through (partial) inbreeding. For root quality traits, carotenoids, and DMCs; amylose-free starch and small starch granules have been reported to have high heritabilities or to depend on single recessive genes. Resistance to pests and diseases (thrips and whiteflies, bacterial blight, super-elongation disease, CMD) and plant architecture traits (erect vs. branching types) have simple inheritance. Certainly another group of traits that would benefit from partial inbreeding are those arising from genetic transformation and gene editing (e.g., herbicide tolerance). Future advances in our knowledge of plant biology (particularly from Arabidopsis) will foster the need and intensity of trait introgression as they are identified in cassava. The reduced within-family variation in progenies derived from partially inbred parents could also contribute toward improvement in more complex traits such as FRY.

Results from this study highlight some key features of cassava breeding. There is a need to shift the current system based on crossing elite germplasm hoping to identify even better progenies, into a system based on the improvement of progenitors with enhanced breeding values, through partial or full inbreeding. This will improve the efficiency of cassava breeding and increase the likelihood of sustained and predictable genetic gains.

## Author contributions

HC implemented the changes in the breeding process that generated the phenotypic data analyzed in this article. He made the analyses and wrote the manuscript; JP was an associate to the breeding program and curated and stored data year after year; OJ conducted a 1-year internship at the program, retrieved the stored data and organized it for its analysis; JL conducted the trials at the sub-humid environment; NM coordinated the production of segregating progenies and the seedling nurseries from which the planting material for the SRT was generated; FC is a senior associate of the program that helped in the overall activities of the program; LP is an assistant in charge of data uploading and curation; CH is the coordinator of the program and also a senior cassava breeding. He reviewed and improved earlier versions of the manuscript.

### Conflict of interest statement

The authors declare that the research was conducted in the absence of any commercial or financial relationships that could be construed as a potential conflict of interest.

## Supplementary material

Supplementary Image **Breeding values for each progenitor**.
