# Supplementary figures and images for: Cassava Breeding I: The Value of Breeding Value
Source: Front Plant Sci. 2016 Aug 29;7:1227. doi: 10.3389/fpls.2016.01227 (PMC5003041; doi:10.3389/fpls.2016.01227)

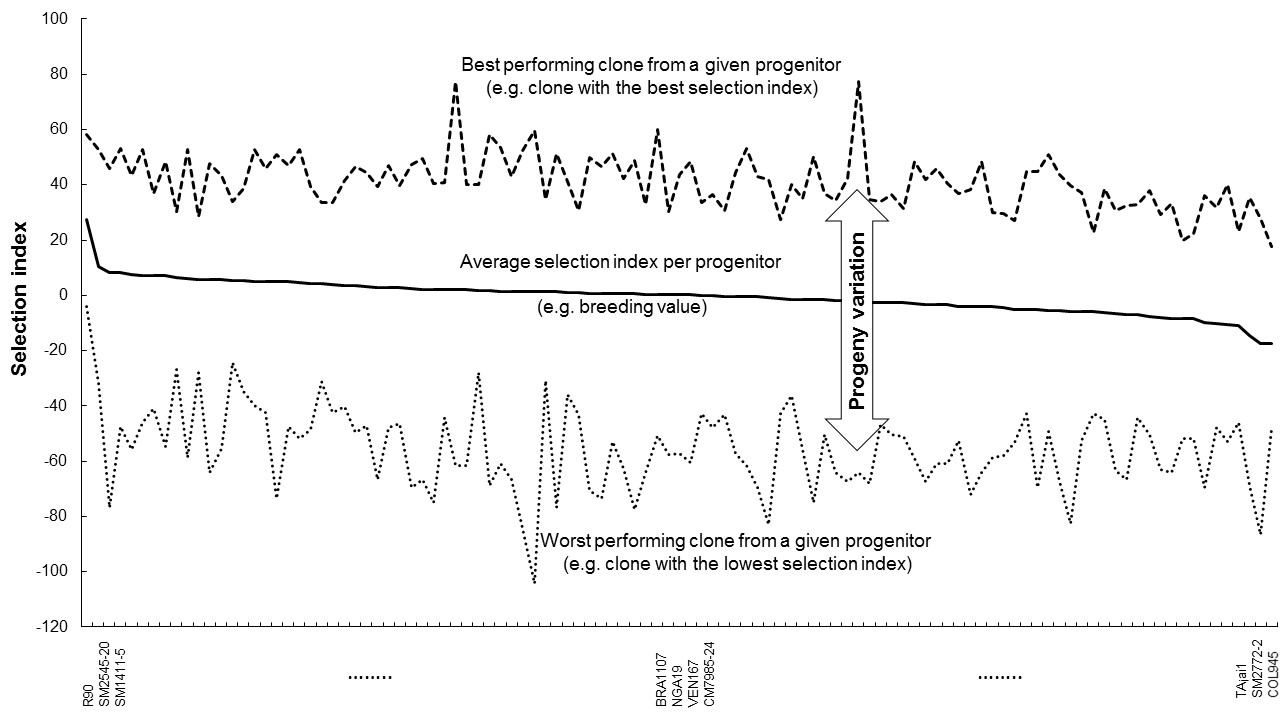

Supplement: Supplementary Image — Breeding values for each progenitor. [file Image1.jpg]
